# Hormonal Regulation and Stimulation Response of *Jatropha curcas* L. Homolog Overexpression on Tobacco Leaf Growth by Transcriptome Analysis

**DOI:** 10.3390/ijms241713183

**Published:** 2023-08-24

**Authors:** Qiuhong Wu, Dongchao Zheng, Na Lian, Xuli Zhu, Jun Wu

**Affiliations:** 1 College of Biological Sciences and Technology, Beijing Forestry University, Beijing 100083, China; hong1481503435@163.com (Q.W.); lianna@bjfu.edu.cn (N.L.); 2 Key Laboratory of Bio-Resources and Eco-Environment of Ministry of Education, College of Life Sciences, Sichuan University, Chengdu 610065, China; dongchao_zheng@scu.edu.cn; 3 National Engineering Research Center of Tree Breeding and Ecological Restoration, Beijing Forestry University, Beijing 100083, China; 4 Sichuan-Chongqing Key Laboratory of Characteristic Biological Resources Research and Utilization, Chengdu 610065, China

**Keywords:** *Flowering locus T*, *Jatropha curcas*, phytohormone, tobacco leaf, transcriptome analysis

## Abstract

The *Flowering locus T* (*FT*) gene encodes the florigen protein, which primarily regulates the flowering time in plants. Recent studies have shown that *FT* genes also significantly affect plant growth and development. The *FT* gene overexpression in plants promotes flowering and suppresses leaf and stem development. This study aimed to conduct a transcriptome analysis to investigate the multiple effects of *Jatropha curcas* L. homolog (*JcFT*) overexpression on leaf growth in tobacco plants. The findings revealed that *JcFT* overexpression affected various biological processes during leaf development, including plant hormone levels and signal transduction, lipid oxidation metabolism, terpenoid metabolism, and the jasmonic-acid-mediated signaling pathway. These results suggested that the effects of *FT* overexpression in plants were complex and multifaceted, and the combination of these factors might contribute to a reduction in the leaf size. This study comprehensively analyzed the effects of *JcFT* on leaf development at the transcriptome level and provided new insights into the function of *FT* and its homologous genes.

## 1. Introduction

The *flowering locus T* (*FT*) gene encodes a unique protein known as florigen, a protein hormone that plays a vital role in promoting flowering in plants [1]. *FT* genes are expressed in the phloem and can be transported to the shoot apices, where they promote the initiation of flowering [2]. The FT protein combines with the basic leucine zipper (bZIP) transcription factor flowering locus D (FD) to form FD/FT heterodimer complexes [3]. These complexes activate the suppression of co overexpression 1 (SOC1) and apetala 1 (AP1) proteins, which play a key role in triggering the transition from vegetative to reproductive growth [4]. Moreover, studies indicated that FT, FD, and 14-3-3 proteins form a flowering activation complex in rice that induces the transcription of downstream flowering-related genes, ultimately leading to the initiation of flowering [5]. So far, the molecular function of FT regulating flowering has been studied thoroughly [6,7].

FT-like proteins have been found to possess other important molecular functions besides their well-known role in regulating flowering. These functions include regulating the growth of shoot meristems and leaves, controlling the formation of callose plugs, modulating the ability of signals to reach the apical meristem, and regulating the transcriptional activity of genes controlling cell division [1,8,9,10]. The overexpression of FT can also regulate H^+^-ATPase activity in guard cells and induce stomatal opening [11]. Florigen in tomatoes has been reported to function as a comprehensive systemic regulator of growth and termination. It controls flowering and also regulates fruit setting, termination of shoot apical meristems, and leaf structures [12]. Furthermore, the ectopic expression of the rice *Hd3a* gene in potatoes revealed that FT could regulate the formation of potato tubers [13]. Interestingly, the *FT* or *FT-like* gene overexpression in multiple species can result in smaller leaves, whereas transgenic plants with inhibited *FT-like* gene expression have larger leaves than wild-type plants [14,15,16,17].

*FT* homologs have been identified in numerous species, indicating their universal role in regulating flowering time [18]. As *FT-like* genes are conserved across plant species and also mediate multiple developmental processes, the molecular plasticity of a single essential gene can drive plant evolution. Moreover, the *FT* gene is associated with environmental adaptability and stress response, potentially regulating plant adaptation to the environment by controlling flowering. Investigating their functions in various plant species can offer valuable insights into plant evolution, diversification, crop domestication, and adaptation mechanisms [10,19].

A comprehensive analysis of the effects of *FT* on leaf development can provide valuable insights into the function of *FT* and its homologous genes, thereby facilitating the development of early maturing crop varieties using *FT* and *FT-like* genes. *JcFT*, an *FT* homolog from *Jatropha curcas* L., has a high sequence similarity with the *FT* of some perennial plants. *JcFT* overexpression in tobacco, *Arabidopsis*, and *J. curcas* has been shown to result in an extremely early flowering phenotype [17,20]. Transgenic tobacco overexpressing *JcFT* exhibits significant changes in the leaf size, which can effectively help investigate the impact of *FT* genes on leaf development [17]. This study aimed to investigate the multiple impacts of *JcFT* overexpression on leaf growth in tobacco using transcriptome analysis. The findings suggested that *JcFT* overexpression influenced various biological processes during leaf development, including plant hormone levels and signal transduction, lipid oxidation metabolism, terpenoid metabolism, and the jasmonic acid (JA)-mediated signaling pathway. This extensive regulation of multiple biological processes can result in morphological changes during leaf development.

## 2. Results

### 2.1. Effect of JcFT^OE^ on Leaf Size and Sampling

**Figure 1:**
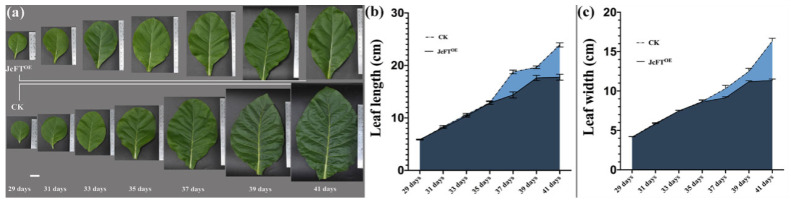
Length and width of leaves of JcFT^OE^ and control plants in different stages. (**a**) Legend of leaves in different stages; bar = 2 cm. (**b**) Length of leaves in different stages; error bar is the standard deviation of the leaf length, *n* = 6. (**c**) Width of leaves in different stages; error bar is the standard deviation of the leaf width, *n* = 6. CK, negative control.

A prominent characteristic of *JcFT* overexpression is the reduction in leaf size [17]. This study continuously measured the length and width of the fourth true leaf of overexpressing *JcFT* (JcFT^OE^) and control plants (Figure 1a). The results indicated no significant difference in the leaf size between JcFT^OE^ and control plants from 29 to 35 days after sowing, but a significant difference was observed after 37 days of sowing (Figure 1b,c). A sudden increase in the leaf size was noted between 35 and 37 days after sowing. The sampling time was chosen to be during the stage of a sudden change in the leaf size, specifically between 35 and 36 days after sowing, to investigate the impact of *JcFT* overexpression on the leaves.

### 2.2. Overview of RNA-Seq Data and Biological Processes Affected by JcFT Overexpression

During the transcriptome analysis of the leaves of three JcFT^OE^ samples and three control samples, 39.90 GB of clean data were obtained (National Center for Biotechnology Information (NCBI) Sequence Read Archive accession number: PRJNA943360). Each sample produced clean data with a size of 6.20 GB, and the Q30 base quality score was greater than 94.15%. Of the clean reads, 93.69–94.35% mapped to the *Nicotiana tabacum* “TN90” genome, with 92.18–94.12% mapped to exons, 3.02–4.41% mapped to introns, and 2.86–3.41% mapped to intergenic regions. The sequencing output data of each sample are presented in Table S1. The Pearson correlation coefficients (*r*) ranged from 0.996 to 0.997 between the three JcFT^OE^ samples and from 0.989 to 0.998 in the three control samples. The *r* values of JcFT^OE^ and control samples were relatively lower (0.958–0.991). The principal component analysis (PCA) results of the fragments per kilobase of exon model per million mapped fragments (FPKM) of each sample showed that the three JcFT^OE^ samples and three control samples were each clustered together (Figure S1), suggesting that sampling and sequencing data produced consistent results.

**Figure 2:**
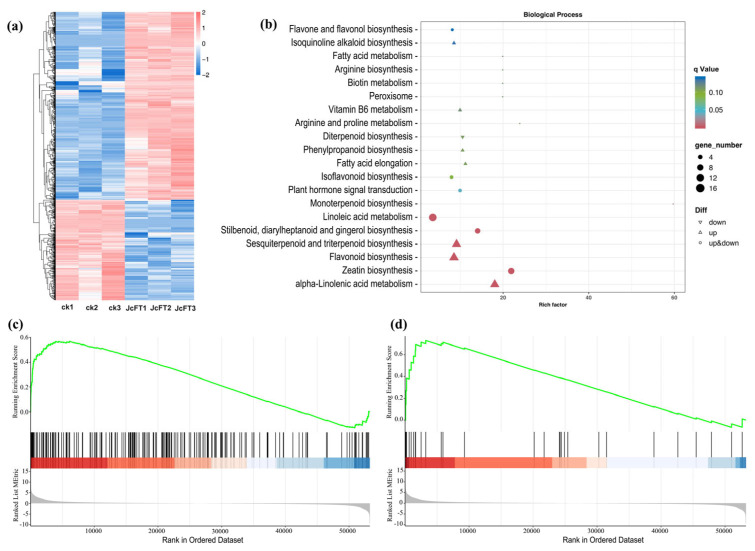
Hierarchical clustering, GO enrichment, and GSEA for DEGs. (**a**) Hierarchical clustering; (**b**) enrichment dot plot of GO biological process of all DEGs; (**c**) GSEA enrichment diagram for DEGs involved in the positive regulation of transfer from RNA polymerase II promoter. The first part is an ES (enrichment score) line plot. The second part displays the gene set member location, with vertical lines marking the positions of gene set members in the sorted gene list. The red region corresponds to genes with high expression in the experimental group, while the blue region corresponds to genes with high expression in the control group. The color intensity represents the expression levels. The third part shows the rank values of all genes after sorting, presented as a gray area plot. (**d**) GSEA enrichment diagram for DEGs involved in positive regulation of transfer from lipid oxidation.

Upon JcFT overexpression, 1119 differentially expressed genes (DEGs) [log2 fold change (FC) ≥ 2 and false discovery rate (FDR) < 0.01] were identified in JcFT^OE^ plants, with 729 upregulated and 390 downregulated (Figure S2). The hierarchical clustering analysis of DEGs demonstrated that the expression pattern of related genes in both the three control samples and the three JcFT^OE^ samples could be accurately distinguished (Figure 2a), indicating that these DEGs well reflected the effect of *JcFT* overexpression. The classification of the Eukaryotic Orthologous Groups (KOG) showed that the DEGs were involved in 25 biological processes (Figure S3). The Gene Ontology (GO) enrichment analysis revealed that *JcFT* overexpression primarily influenced various processes. These processes included positive regulation of transfer from RNA polymerase II promoter, lipid oxidation, oxylipin biosynthetic process, regulation of defense response, response to wounding, and regulation of JA-mediated signaling pathway, among which genes involved in the last three processes were all upregulated (Figure 2b). Additionally, according to gene set enrichment analysis (GSEA), genes involved in the positive regulation of transfer from RNA polymerase II promoter and lipid oxidation tended to be upregulated (Figure 2c,d).

**Figure 3:**
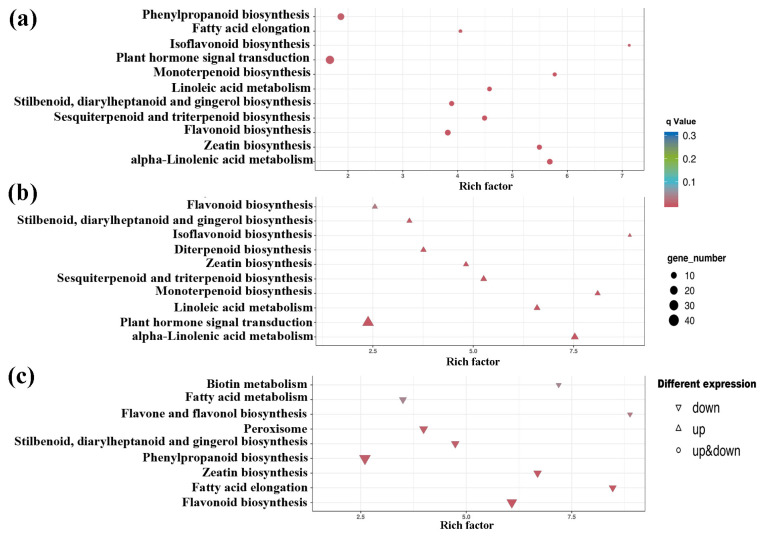
KEGG pathway enrichment analysis for DEGs. KEGG pathway enrichment dotplots of (**a**) all DEGs; (**b**) upregulated DEGs; (**c**) downregulated DEGs.

With respect to the Kyoto Encyclopedia of Genes and Genomes (KEGG) enrichment analyses for DEGs, 11 pathways exhibited significant effects. These pathways included plant hormone signal transduction, flavonoid biosynthesis, zeatin biosynthesis, phenylpropanoid biosynthesis, fatty acid elongation, and alpha-linolenic acid metabolism, among others (Figure 3a). The KEGG enrichment analysis for upregulated and downregulated DEGs revealed significant upregulation of the plant hormone signal transduction pathway, diterpenoid and monoterpenoid biosynthesis, and linoleic acid metabolism (Figure 3b). Conversely, fatty acid metabolism, fatty acid elongation, and phenylpropanoid biosynthesis were enriched in downregulated DEGs (Figure 3c). Notably, both upregulated and downregulated DEGs were significantly enriched in zeatin biosynthesis and flavonoid biosynthesis (Figure 3b,c).

The DEGs were further analyzed using ClueGO in the Cytoscape (v3.8.2) software (showing only pathways with *p* ≤ 0.05). Twelve functional groups of GO terms or KEGG pathways were identified, and each group was represented by its most significant term (Table 1 and Figure S4), including the regulation of the JA-mediated signaling pathway, oxylipin metabolism, hormone level regulation, isoprenoid biosynthesis, carboxy-lyase activity, carbon–oxygen lyase activity, response to wounding and detoxification, among others. The number of DEGs and percentage of GO terms included in each group are presented in Table 1, and specific GO terms for each group are depicted in Table S2. Among these groups, the regulation of the JA-mediated signaling pathway had the most terms associated with it, with seven terms accounting for 33.33% and 84 DEGs. These identified functional groups suggested that *JcFT* overexpression significantly impacted the hormone-related pathways in leaves, affecting the hormone levels, particularly the metabolism and response of JA.

### 2.3. Regulation of Cytokinin and Auxin Levels

**Figure 4:**
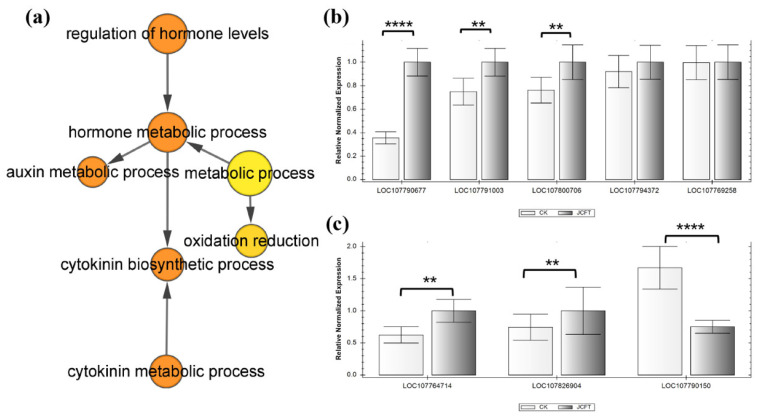
GO terms and RT-qPCR analysis of DEGs related to the regulation of hormone levels, and the results tested by *p*-value. (**a**) Overrepresented GO terms annotated by BiNGO [the color of a node represents the corrected *p*-value, with the scale ranging from yellow (*p* = 0.01) to dark orange (*p* = 0.01 × 10^−5^), and the size of a node indicates the number of genes involved]. (**b**,**c**) RT-qPCR analysis of DEGs involved in cytokinin and auxin metabolic processes related to the regulation of hormone levels, respectively. Bars represent gene expression mean and standard error of the mean, *n* = 3, ** *p* < 0.01, **** *p* < 0.0001. LOC107800706: *CKX1*; LOC107794372 and LOC107769258: *LOG3*; LOC107791003 and LOC107790677: *CKX5*; LOC107764714, LOC107790150, and LOC107826904: *ILL2*, *ILL3*, and *ILL4*, respectively.

A group related to the regulation of hormone levels was significantly annotated through the analysis of DEGs using ClueGO (Table 1). Further examination of these DEGs using BiNGO revealed that the regulation of hormone levels was primarily linked to cytokinin and auxin metabolic processes, where oxidation-reduction played a crucial role (Figure 4a). The impact on cytokinin metabolic processes involved three significantly upregulated *cytokinin dehydrogenase* (*CKX*) and *two lonely guy* (*LOG*) gene families, while the auxin metabolic process involved three indole-3-acetic acid (IAA) amino acid hydrolase *ILR1-like* (*ILL*) gene families, and two of them were upregulated (Table S3). Moreover, the reverse transcription-quantitative polymerase chain reaction (RT-qPCR) analysis confirmed that *JcFT* overexpression led to changes in the expression of these eight genes, which was consistent with the findings of the transcriptome analysis (Figure 4b,c). CKX, one of the key enzymes regulating CK levels in plants, caused irreversible cytokinin degradation [21], indicating that the cytokinin levels of JcFT^OE^ leaves were likely reduced.

### 2.4. Regulation of Plant Hormone Signal Transduction

**Figure 5:**
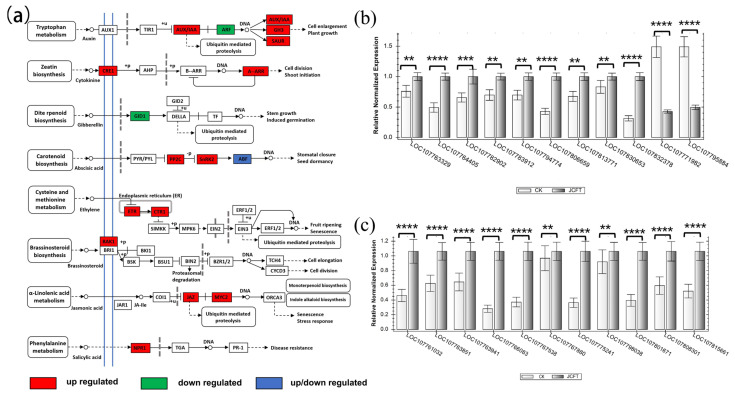
KEGG pathway map of plant hormone signal transduction of DEGs and the RT−qPCR analysis of some key genes. (**a**) Color displays the nodes of DEGs in plant hormone signal transduction pathways. (**b**) RT−qPCR analysis of DEGs involved in JAZs. (**c**) RT−qPCR analysis of DEGs involved in AUX/IAA, SAUR, GH3, and ARF. Bars represent gene expression means and standard errors of the means, *n* = 3, ** *p* < 0.01,*** *p* < 0.001, **** *p* < 0.0001. LOC107832378: SAUR50; LOC107830653 and LOC107764405: *IAA14*; LOC107782902, LOC107813771, and LOC107763329: *auxin-induced protein 15A*; LOC107806659: *AUX22D*; LOC107771982 and LOC107795884: *ARF9*; LOC107794774: *GH3.10*; LOC107783912: *SAUR21*; LOC107815661 and LOC107801671: *TIFY10A*; LOC107808301, LOC107798038, and LOC107763851: *TIFY10B*; LOC107775241, LOC107767538, and LOC107766083: *TIFY6B*; LOC107767880 and LOC107763941: *JAZ7*; and LOC107761032: *TIFY4B*.

The FT protein is a proteinaceous hormone [1]. The KEGG pathway annotation showed that *FT* gene overexpression affected numerous biosynthesis pathways, metabolic pathways, and plant hormone signal transduction, which are relevant to eight plant hormone signal transduction pathways, including auxin, cytokinin, gibberellin, abscisic acid (ABA), ethylene, brassinosteroid, JA, and salicylic acid (SA). Transcriptome data indicated that all but 3 of the 17 pathways involved had upregulated DEGs associated with them (Figure 5a). This suggested that the transcription of phytohormone signal transduction factors in JcFT^OE^ plants was enhanced.

Of these pathways, the JA signal transduction pathway was found to be the most significantly affected, with a large number of DEGs involved, including 13 jasmonate zim domain (JAZ) and 4 basic helix-loop-helix (bHLH) transcription factor myelocytomatosis protein 2 (MYC2), all of which were significantly upregulated (Table S4). The expression patterns of these DEGs, as identified in the transcriptome analysis, were further validated by RT-qPCR analysis (Figure 5b), which confirmed that JcFT overexpression significantly impacted the expression of genes related to JA signal transduction.

The signal transduction pathway of auxin involved four types of DEGs, namely three *auxin/indole-3-acetic acid* (*AUX/IAA*) genes, one *Gretchen Hagen 3* (*GH3*) gene, seven *small auxin up RNA* (*SAUR*) genes, and four *auxin response factor* (*ARF*) genes. Except for the downregulation of *ARF* expression, the expression of other factors was upregulated. RT-qPCR analysis further confirmed the expression of these DEGs, which was consistent with the results of transcriptome analysis (Figure 5b).

The cytokinin signaling pathway in JcFT^OE^ plants was found to be associated with one cytokinin receptor 1 (CRE1) and two type-A authentic response regulators (ARRs). CRE1 transduces cytokinin signals across the membrane, which is a class of plant hormones that plays an important role in regulating cell division and differentiation [22]. The proteins encoded by ARRs act as negative regulators of cytokinin response, and mutations in these genes have been shown to increase cytokinin sensitivity [23]. Therefore, the leaves in the JcFT^OE^ group were less sensitive to cytokinins than those in the control group.

The signaling pathways related to gibberellin and ABA showed distinct responses in JcFT^OE^ plants. The gibberellin signaling pathway was primarily associated with a downregulated gene, *gibberellin-insensitive dwarf 1* (*GID1*), which is crucial for the positive regulation of gibberellin signals. On the contrary, the DEGs of the ABA signal transduction pathway were associated with two clade A protein phosphatases type-2C, three sucrose non-fermenting 1-related subfamily 2 (SnRK2), and two ABA-responsive element (ABRE) binding factors (ABFs). The SnRK2 protein kinase plays a key role in regulating the transcriptional response to ABA. The upregulation of SnRK2 suggested that the response to ABA was likely to increase in the leaves of JcFT^OE^ plants.

The DEGs involved in the ethylene signal transduction pathway included one upregulated ethylene insensitive 4 (EIN4) and one upregulated constitutive triple response 1 (CTR1). EIN4 is an ethylene receptor that transmits signals to downstream CTR1, a protein kinase that negatively regulates ethylene signaling and suppresses ethylene responses in *Arabidopsis* [24,25]. The SA signal transduction pathway involves the nonexpresser of pr genes 1, which is a receptor of SA and plays a role in regulating SA-induced defense gene expression [26].

In summary, in plant signal transduction, JcFT^OE^ affected the sensitivity of various phytohormones, including auxin, cytokinin, gibberellin, ABA, ethylene, JA, and SA. This was evidenced by the significant upregulation of DEGs associated with these pathways and the increased expression of related transcription factors. Additionally, the transmembrane transport capacity of cytokinin and brassinosteroid in leaves was also impacted by JcFT^OE^.

### 2.5. Effects of JcFT^OE^ on Lipid Biosynthetic Process

**Figure 6:**
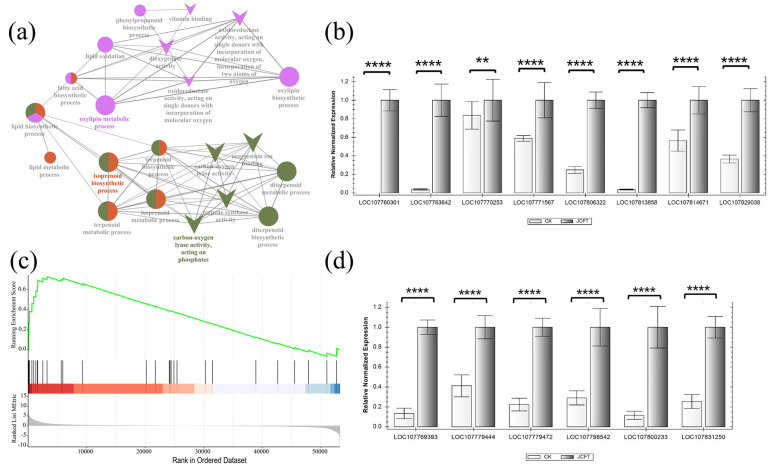
Processes and molecular functional groups related to lipid metabolism enriched in DEGs, GSEA analysis of linoleic acid metabolism, and RT–qPCR analysis of key genes. (**a**) Oxylipin metabolic process, isoprenoid biosynthetic process, and carbon-oxygen lyase activity of DEGs enriched by ClueGO (color shows the different biological process or molecular function groups, circle indicates the biological process, and V indicates the molecular function). (**b**) RT–qPCR analysis of eight upregulated oxylipin metabolic processes related to DEGs. (**c**) GSEA enrichment diagram of DEGs involved in linoleic acid metabolism. (**d**) RT–qPCR analysis of six upregulated isoprenoid biosynthetic processes related to DEGs. Bars represent gene expression mean and standard error of the mean, *n* = 3, ** *p* < 0.01, **** *p* < 0.0001. LOC107829038: *LOX6*; LOC107814671 and LOC107771567: *LOX3.1*; LOC107813858: *DOX1*; LOC107806322: *LOX1.5*; LOC107770253: *LOX1.5*; LOC107763642: *LOX2.1*; LOC107760301: *LOX1.6*; LOC107831250: *(−)-alpha-terpineol synthase*; LOC107800233 and LOC107779472: *alpha-farnesene synthase*; LOC107798542: *(R)-linalool synthase TPS5*; LOC107769393: *viridiflorene synthase*; and LOC107779444: *heterodimeric geranylgeranyl pyrophosphate synthase small subunit*.

In the ClueGO analysis of DEGs, the process groups of the oxylipin metabolic process and isoprenoid biosynthetic process were significantly enriched (Figure 6a), indicating that JcFT overexpression had a significant impact on the two interrelated processes of lipid biosynthesis in tobacco leaves. The DEGs involved in the oxylipin metabolic process were found to be the same as those in the oxylipin biosynthetic process. These DEGs were related to dioxygenase activity, oxidoreductase activity, and other functional groups. Similarly, DEGs in the enriched isoprenoid biosynthetic process were related to the functional group of carbon-oxygen lyase activity, acting on phosphates.

Oxylipins, which are derived from the oxidation of polyunsaturated fatty acids such as linoleic acid, are enzymatically converted by lipoxygenase (LOX) or α-dioxygenases (α-DOX) [27]. These enzymes play a role in plant signaling responses to both biotic and abiotic stresses [28]. In this study, the DEGs related to oxylipin metabolism included eight LOXs and one α-DOX, all of which were oxygenase genes involved in linoleic acid metabolism (Table S5). The *LOXs* comprised one *LOX6*, one *LOX1.6*, two *LOX1.5*, two *LOX3.1*, and two *LOX2.1* genes. All but one, *LOX2.1*, were upregulated (the log_2_FC value increased from 1.17 to 6.10).

**Figure 7:**
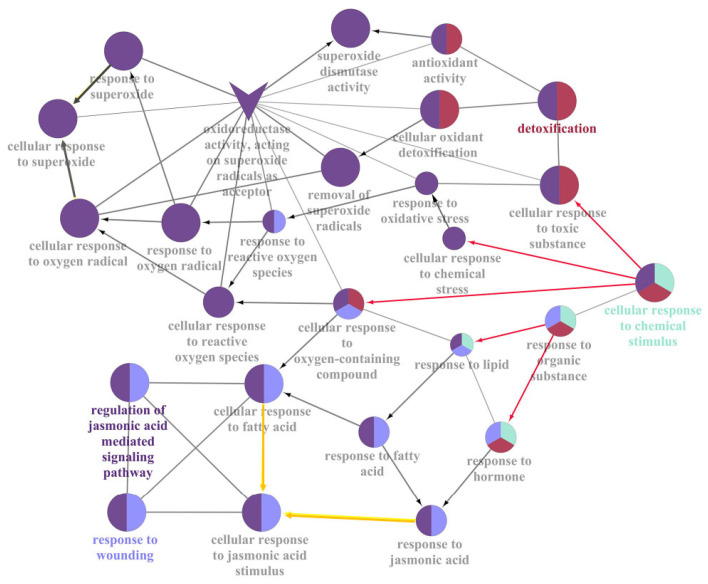
Overrepresented GO terms of cellular response to chemical stimulus in DEGs annotated by ClueGO (directed edges indicate directed ontology relations and undirected edges indicate participation together in a biological process but without a clear upregulated or downregulated relationship).

The GSEA suggested that the transcription of DEGs involved in linoleic acid metabolism tended to be upregulated (Figure 6b). Furthermore, RT-qPCR analysis of these upregulated DEGs confirmed the significant changes in their expression (Figure 6c). These results indicated that *JcFT* overexpression in tobacco leaves significantly increased the expression of genes related to linolenic acid oxidation, which was associated with increased oxylipin content. As linolenic acid is a precursor in JA biosynthesis [29], the upregulated *LOXs* and *DOX1* may affect the JA-related pathways (Figure 7).

The isoprenoid biosynthetic process involved 18 DEGs, of which 14 were upregulated. Six randomly selected upregulated genes were subjected to RT-qPCR analysis to validate the transcriptional analysis results, which showed consistent results (Figure 6d). These genes were involved in the biosynthesis of monoterpenoid, sesquiterpenoid, triterpenoid, terpenoid backbone, and carotenoid (Table S6). Notably, some genes, such as *(−)-alpha terpineol synthase* (LOCA107831250), *viridiflorene synthase* (LOC107769393), and *alpha-farnesene synthase* (LOC107800232), were significantly upregulated (all log_2_FC > 6), indicating that *JcFT* overexpression caused increased expression of genes related to oxylipins and isoprenoid synthesis in tobacco leaves.

### 2.6. Stimulus Response Induced by JcFT Overexpression

In the ClueGO analysis, several significantly enriched and interrelated groups were identified, including the regulation of the JA-mediated signaling pathway, response to wounding, cellular response to chemical stimulus, and detoxification (Table 1 and Figure S4). The cellular response to the chemical stimulus was further classified into four aspects: cellular response to oxygen-containing compounds, cellular response to organic substances, cellular response to toxic substances, and cellular response to chemical stress (Figure 7, indicated by red arrows). Oxygen-containing compounds included fatty acids and superoxide, while organic substances mainly comprised lipids (primarily fatty acids) and hormones (primarily JAs). Detoxification of toxic substances and chemical stress involves the removal of superoxide radicals and superoxide dismutase activity. Therefore, *JcFT* overexpression in tobacco leaves resulted in a stimulated response to JA, fatty acids, and superoxide. This was primarily reflected in GO terms related to the JA-mediated signaling pathway, response to wounding, cellular response to chemical stimulus, and detoxification.

The DEGs involved in response to fatty acids were found to be the same as those involved in response to JA, thus indicating a response to JA stimulation (Figure 7, indicated by yellow arrows). Further analysis revealed that *JcFT* overexpression led to an increase in the expression of *LOX-related* genes, which caused the formation of oxylipins, an oxygen-containing compound, and eventually triggered a JA reaction. This response to JA involved 11 upregulated DEGs, including 9 *TIFY* (threonine, isoleucine, phenylalanine, tyrosine) genes (6B, 4B, 10B, and 10A) and 2 *JAZ7*, which play critical roles in plant growth and stress response as repressors of jasmonate responses [30,31]. Notably, 10 of the 11 JA-related DEGs were also JAZ-related DEGs of the plant hormone signal transduction pathway (Tables S4 and S7). The qPCR analysis further validated the upregulation of the DEGs involved in response to JA in the leaves of JcFT^OE^ plants (Figure 5c). These results suggested that the response of tobacco leaves to JA-like stimulation, induced by *JcFT* overexpression, was mainly reflected in the signal transduction pathways.

## 3. Discussion

The mechanism underlying the FT-protein-induced flowering has been extensively studied [6,32]. Although *FT* overexpression usually leads to early flowering in plants, few *FT* genes have been used to produce early maturing varieties for seed harvesting, largely due to the negative impact of *FT* overexpression on plant growth, including stem thinning and reduced leaf number and size, which ultimately affects seed yield [33]. This study performed a transcriptome analysis of tobacco leaves induced by *JcFT* overexpression to elucidate the effects of *FT* on leaf development. The results indicated that *JcFT* overexpression significantly altered hormone signal transduction, lipid oxidation metabolism, and terpenoid metabolism during leaf development. Moreover, the transcription of genes related to the JA pathway and cell division also underwent changes, ultimately leading to a decrease in the leaf size. This result was consistent with previous studies on *FT* of *Nicotiana tabacum* (*NtFT*) in tobacco and provided further evidence for conserving *FT-like* genes in various plant species [34].

The leaf size of JcFT^OE^ tobacco was significantly smaller than that of the control (Figure S5a,b). A comparison of leaf epidermal cells confirmed that JcFT^OE^ tobacco leaves had larger epidermal cells and fewer cell numbers than the control leaves (Figure S5c,d). The expression of *SAUR*s and *CKX*s in JcFT^OE^ tobacco leaves was significantly altered (Figure 4b and Figure 5a,b). SAUR proteins regulate protein phosphatases to control H^+^-ATPase activity and play a central role in auxin-induced acid growth [35]. *SAUR* overexpression increases the phosphorylation and activity of plasma membrane H^+^-ATPase, leading to cell wall acidification and promoting cell expansion [36,37]. In this study, a significant upregulation of *SAUR* expression in JcFT^OE^ tobacco leaves suggested enhanced cell expansion. Kinoshita et al. demonstrated that *FT* could activate plasma membrane H^+^-ATPase in guard cells and is believed to be a general growth regulator by modulating H^+^-ATPase activity [11]. This study speculated that the increased expansion of leaf epidermal cells caused by *FT* overexpression might be due to the activation of H^+^-ATPase by increasing the *SAUR* expression. CKX is one of the key enzymes regulating the CK level in plants, which causes irreversible degradation of cytokinin [21]. The significant upregulation of *CKX* expression in JcFT^OE^ tobacco leaves indicated a decrease in cytokinin levels in JcFT^OE^ leaves, which might be an essential factor in reducing cell numbers. The JAZ proteins act as repressors of the JA signaling pathway. The transcription factor MYC2 plays a crucial role in the activation of JA response genes and is considered the “Master Switch” of the jasmonate signaling pathway [38,39]. In the absence of JA, MYC2 functions as a transcriptional repressor due to physical interaction with the members of the JAZ family [30]. However, when JA levels are elevated, the rate of JAZ turnover increases, leading to the release of MYC2 from repression. Bioactive JAs promote the interaction between JAZ protein and the coronatine insensitive 1 (COI1) component of the SCFCOI1 ubiquitin E3 ligase, resulting in the ubiquitination and degradation of JAZ by the 26S proteasome [40,41]. As a result, elevated levels of JA trigger MYC2 to release from the repression caused by JAZ interaction, resulting in the activation of JA response genes. The increased transcription of *MYC2* and *JAZ* in JcFT^OE^ plants might enhance the sensitivity of plants to jasmonate signaling.

Some researchers described florigen as a protein hormone [1], so its relationship with and influence on other plant hormones are essential topics. Combining the research on the effect of *JcFT* overexpression on tobacco stem growth [17] and the results of this study, it was found that *JcFT* overexpression had an impact on almost all types of hormone signaling pathways in tobacco stems and leaves, such as auxin, cytokinin, gibberellin, ABA, and SA. This suggested that *FT* and *FT-like* gene overexpression generally affected the signaling pathways of multiple hormones. Among these, the auxin-related signaling pathway involved more genes in both stems and leaves, most of which were significantly upregulated *SAUR* genes (7–30 genes). Additionally, the expression of response to *JA* genes involved in the JA signaling pathway was significantly increased in leaves but not in stems. Moreover, gene expression involving carbohydrates, such as hemicellulose and cellulose, was significantly affected in stems, while gene expression involving the lipid synthesis pathway was significantly affected in leaves. Wu et al. also showed that *JcFT* overexpression had a comprehensive impact on the tobacco stem at multiple levels of the cell wall, cytoplasm, and nucleus, including the core oscillators involving circular rhythm, cell cycle regulation, initiation of DNA replication, and expression of genes related to xylem development. The initiation of DNA replication and cell cycle regulation might be one of the main factors for the thinning of *JcFT*-overexpressed tobacco stems [17]. In this study, *JcFT* overexpression also affected multiple pathways in tobacco leaves but did not exhibit multidimensional effects as in stems. This might be related to the reactions of different tissues or to the period of material collection. This might be related to the reactions of different tissues or the time of material collection, with leaf collection occurring earlier.

In this study, *JcFT* overexpression in tobacco was achieved through genetic transformation, and the insertion of the *JcFT* gene into the tobacco genome may also have an impact on transcription, which is not the effect of *JcFT*. If the used transgenic independent lines would involve the insertion of the same functional gene, the result may reflect the functions of the inserted genes.

*FT* gene overexpression significantly affects the development of tobacco roots, stems, and leaves, besides their well-known function of promoting flowering [42,43,44,45,46]. Inhibition of leaf growth appears to be a fundamental effect of the *FT* gene, which has only evolved in seed plants. The FT protein is usually expressed in companion cells and then transported through the phloem to the shoot apical meristem to promote flowering [47]. However, when plants overexpressing *FT* are grafted onto an *ft* mutant or a wild-type plant, the ft or wild-type plants bloom earlier without undergoing visible changes such as those seen in *FT*-overexpressed plants [12,43,48,49]. These observations demonstrated that vascular bundle transport can bypass the effects of *FT*, resulting in the shrinking of leaves. When *FT* is naturally expressed, transported FT proteins may appear in cells other than companion cells, phloem, and stem apex cells. Thus, most cells in roots, stems, and leaves avoid the effects of *FT*, such as inhibiting cell division. As a result, although *FT* promotes flowering, plants have evolved unique expression and transportation mechanisms to avoid the influence of *FT* on most plant cells and tissues. Therefore, *FT* initiates reproduction, but plants prevent its negative effects on vegetative growth.

## 4. Materials and Methods

### 4.1. Vector Construction, Plant Transformation, and Cultivation

Li et al. [20] reported that the *JcFT* gene (accession no. NM_001308752) was overexpressed in *N. tabacum* “SR1.” The *JcFT* sequence, isolated from *J. curcas*, was inserted into the *SmaI* and *SacI* sites of the binary vector pBI121 (DNA Cloning Service, Genewiz, Suzhou, China). The resulting vector was designated as CaMV 35S::JcFT, with an empty vector serving as the negative check (CK). The constructed vectors were introduced into the *Agrobacterium tumefaciens* strain EHA105 and then transformed into the wild-type *N. tabacum* “SR1” using a leaf disk method [50]. Transgenic plants were identified through PCR amplification of *aminoglycoside 3′-phosphotransferase* (*NPTII*) and *JcFT* genes, using leaf DNA as a template. The primers for *NPTII* and *JcFT* amplification are depicted in Table S8. The plants were grown under a 16 h light/8 h dark photoperiod at 26 °C. Transgenic seeds from the T5 generation with randomly selected four independent lines were harvested and used for this study. The JcFT^OE^ plants were identified by morphological characteristics and one-step RT-PCR analysis using leaf total RNA as a template through the HiScript II One-Step RT-PCR Kit (Vazyme, Nanjing, China) (Figure S6). The length and width of the fourth true leaf were recorded daily from 29 to 41 days of sowing.

### 4.2. RNA Isolation, Library Construction, and Sequencing

*JcFT* transgenic tobacco and control tobacco were simultaneously sown with 80 seeds each. Subsequently, transgenic plants were distinguished based on morphological characteristics and RT-PCR analysis. Select three out of four JcFT^OE^ independent lines, each corresponding to a duplicate. A total of 20 *JcFT* transgenic plants, with each line consisting of 6–7 plants and an equal number of control plants, were prepared for further experimentation. On the 33rd and 35th days of sowing, 2–3 plants were randomly selected from each line to collect the fourth true leaf of each plant for transcriptome analysis. Three replicates each of JcFT^OE^ and control were established, consisting of 2–3 mixed and ground leaves for RNA extraction.

Total RNA was extracted using TaKaRa MiniBEST Plant RNA Extraction reagents following the manufacturer’s protocol (TaKaRa Bio, Inc., Dalian, China). The quality and concentration of RNA were measured using a NanoDrop ND-1000 spectrophotometer (Thermo Fisher Scientific, Dover, DE, USA) and an Agilent 2100 Bioanalyzer (Agilent Technologies, Santa Clara, CA, USA), respectively. A NEBNext Poly(A) mRNA Magnetic Isolation Module (E7490; NEB, Rowly, MA, USA) was used to isolate mRNA, which was then fragmented into approximately 200-nt RNA inserts. Next, double-stranded cDNA was synthesized from these fragments, and end-repair/dA-tail and adaptor ligation were performed. Suitable fragments were isolated using Agencourt AMPure XP beads (Beckman Coulter, Inc., Brea, CA, USA) and subsequently enriched through PCR amplification. The amplification procedure consisted of an initial denaturation at 98 °C for 30 s, 14 cycles of denaturation at 98 °C for 10 s, annealing at 65 °C for 30 s, and extension at 72 °C for 30 s, followed by a final extension at 72 °C for 5 min, and the process concluded with a 4 °C hold. Finally, the cDNA libraries of tobacco stems were sequenced by an Illumina HiSeq 2500 sequencing platform (Illumina, Inc., San Diego, CA, USA) using a flow cell.

### 4.3. Global and Differential Gene Expression Analysis of RNA-Seq Data

The reads were initially filtered to obtain clean reads by removing reads containing linkers and those of low quality, defined as reads with a proportion of undetermined bases exceeding 10% or with more than 50% of bases having a quality score ≤ 10, so as to ensure the accuracy of subsequent analyses [51]. The clean reads were then mapped to the tobacco cultivar TN90 genome (https://www.ncbi.nlm.nih.gov/bioproject/208209, accessed on 29 May 2014) using the HISAT2 program [52]. StringTie software (v1.3.3b) [53] was used to construct transcripts and evaluate gene expression. The Pearson correlation analysis was conducted on the expression levels of paired samples [54], and the coefficient of correlation (*r*) was used to evaluate the correlation strength. BLAST (v2.10.0) software [55] and various databases, including NR (non-redundant protein sequence database), Swiss-Prot, GO, COG, KOG, Pfam, and KEGG, were employed to annotate the genes. DEGseq [56] was used to identify DEGs between sample groups, according to the screening criteria of log_2_ FC ≥ 2 and FDR < 0.01. The results of differential expression analysis and the interaction pairs included in the STRING database [57] were integrated to construct a DEG interaction network, which was then imported into Cytoscape version 3.8.1 [58] for visual analysis. ClueGO was used to further analyze the functional grouping network of terms or pathways of the DEG sets, showing only pathways with *p* ≤ 0.05 [59]. The overrepresented GO terms in the network were identified and displayed as a network of significant GO terms using BiNGO with a significance level of 0.05 [60].

### 4.4. RT-qPCR Analysis

Total RNA was extracted from fresh plant tissue using a plant RNA extraction kit (Chengdu Biofit Biotechnologies Co., Ltd., Chengdu, China). First-strand cDNA was synthesized according to the manufacturer’s protocols, using the HiScript III RT SuperMix for qPCR Kit with gDNA wiper (Vazyme). RT-qPCR analysis was performed using the AceQ qPCR SYBR green master mix (Vazyme) on a CFX Connect Real-Time System (Bio-Rad, Hercules, CA, USA). The primers used in RT-qPCR and RT-PCR are presented in Table S8. Two independent biological replicates and three technical replicates were used for each sample, and data were analyzed using the 2^−ΔΔCT^ method [61]. The *L25 ribosomal* and *Elongation factor 1a* genes were used as internal reference genes [62] to ensure the accuracy of subsequent analyses.

## Figures and Tables

**Table 1:** Functional groups of GO terms or KEGG pathways identified by ClueGO.

Label of the Most Significant Term Per Group	Amount of DEGs	% Terms Per Group
Regulation of JA-mediated signaling pathway	84	33.33
Response to wounding	78	15.69
Carbon-oxygen lyase activity, acting on phosphates	68	10.78
Detoxification	91	9.8
Oxylipin metabolism	75	9.8
Isoprenoid biosynthetic process	71	6.86
Regulation of hormone levels	21	3.92
Oxidoreductase activity, acting on paired donors, with incorporation or reduction of molecular oxygen	55	3.92
Cellular response to chemical stimulus	86	3.92
Carboxy-lyase activity	11	0.98
UDP-glycosyltransferase activity	26	0.98

Note: % terms per group refers to the proportion of annotated terms to the total terms in each group.

## Data Availability

The raw sequence reads have been deposited in the National Center for Biotechnology Information under accession number PRJNA943360. These can be accessed at https://www.ncbi.nlm.nih.gov/bioproject/PRJNA943360, accessed on 11 March 2023.

## Supplementary Materials

The following supporting information can be downloaded at: https://www.mdpi.com/article/10.3390/ijms241713183/s1.
