# Supplementary material for: Hormonal Regulation and Stimulation Response of Jatropha curcas L. Homolog Overexpression on Tobacco Leaf Growth by Transcriptome Analysis
Source: Int J Mol Sci. 2023 Aug 24;24(17):13183. doi: 10.3390/ijms241713183 (PMC10487882; doi:10.3390/ijms241713183)
Supplement: Supplementary file 1 [file ijms-24-13183-s001.zip › ijms-2480503-supplementary.pdf]

1 Supplementary Figures

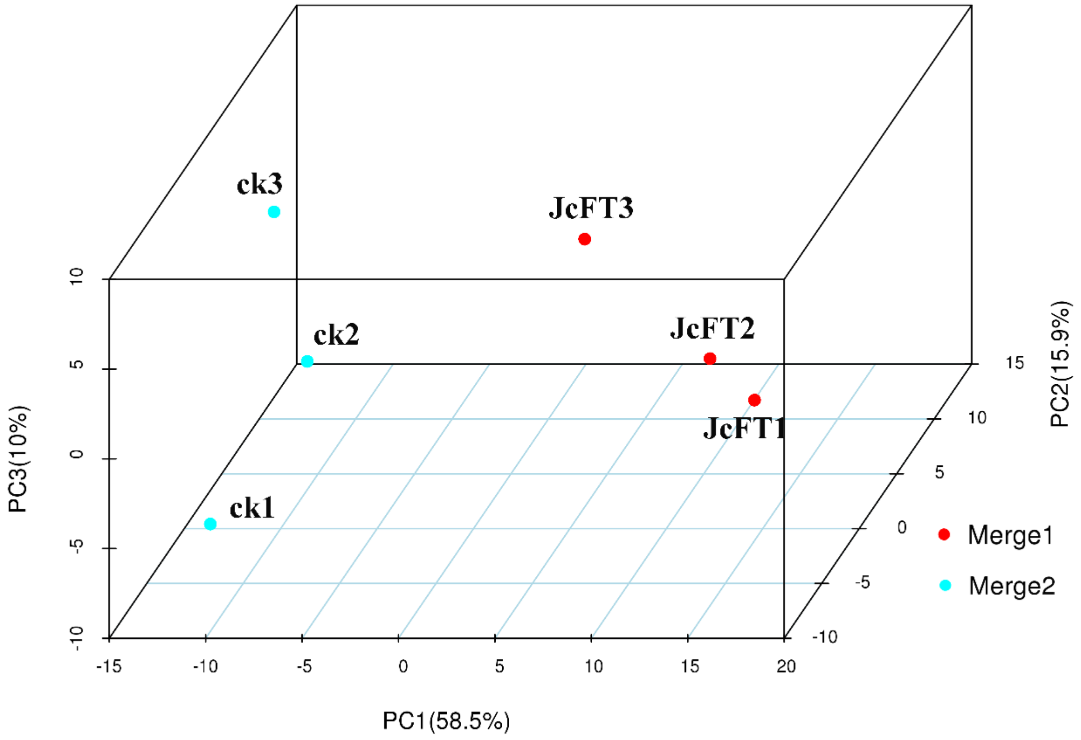

Figure S1. Principal component analysis (PCA) Plot of each sample's FPKM (PC1, PC2 and PC3 are three principal components; different colors represent different groups of biological replicates. FPKM, Fragments Per Kilobase of transcript per Million fragments mapped)

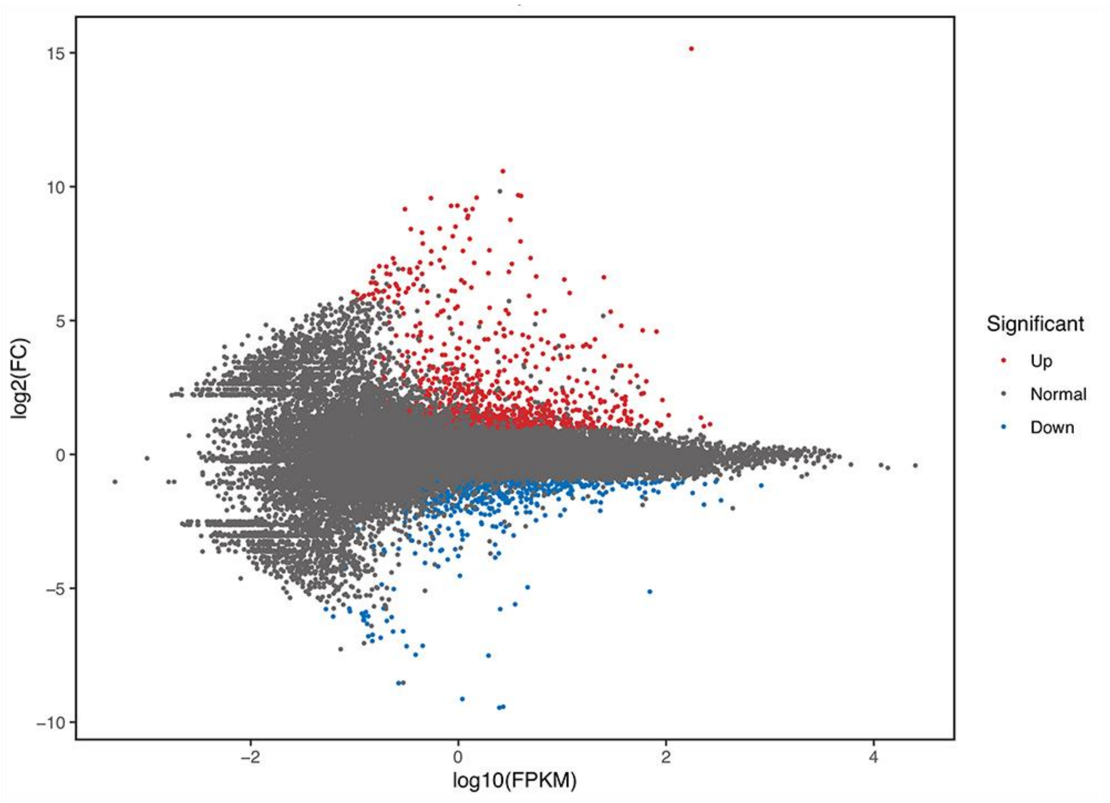

Figure S2. M-versus-A plot of differentially expressed genes between JcFT^{OE} and control samples. (Each dot represents a gene. FPKM = (Number of reads mapped to a gene/Gene length) *1000000/Total mapped reads, FC: Fold Change)

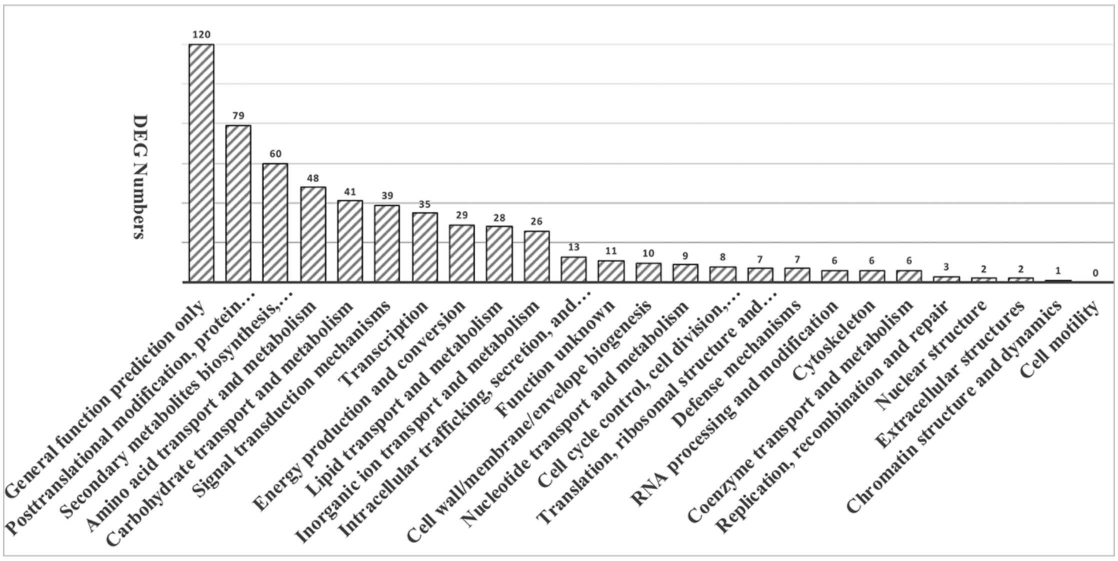

Figure S3. KOG (Eukaryotic Orthologous Groups classification) enrichment of differentially expressed genes

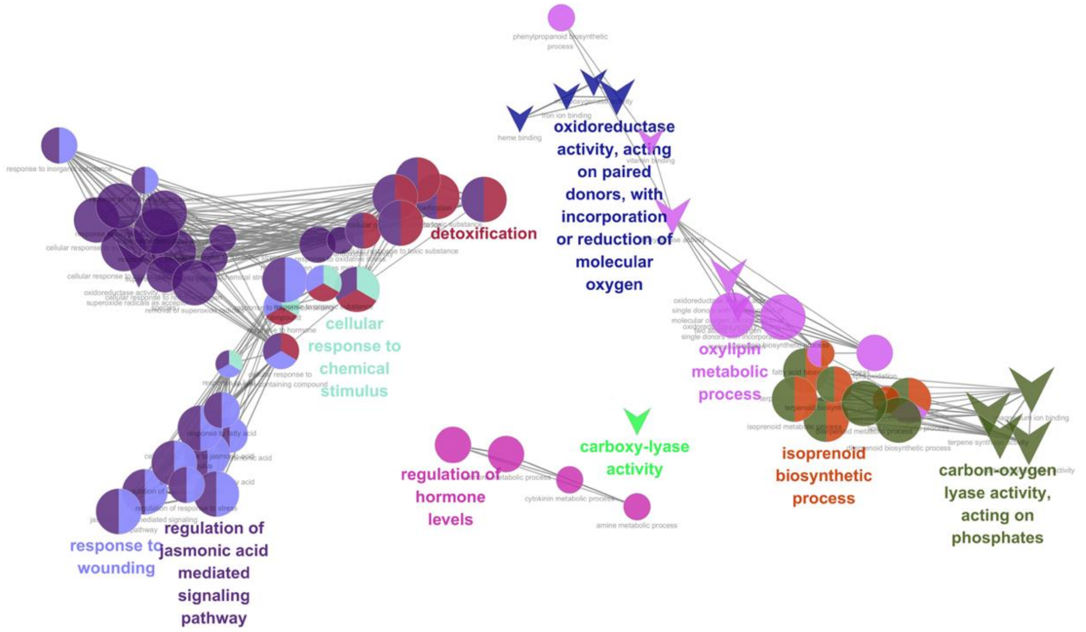

Figure S4. ClueGO grouping network of differentially expressed genes (Each circle represents biological process, and each V shape represents molecular function)

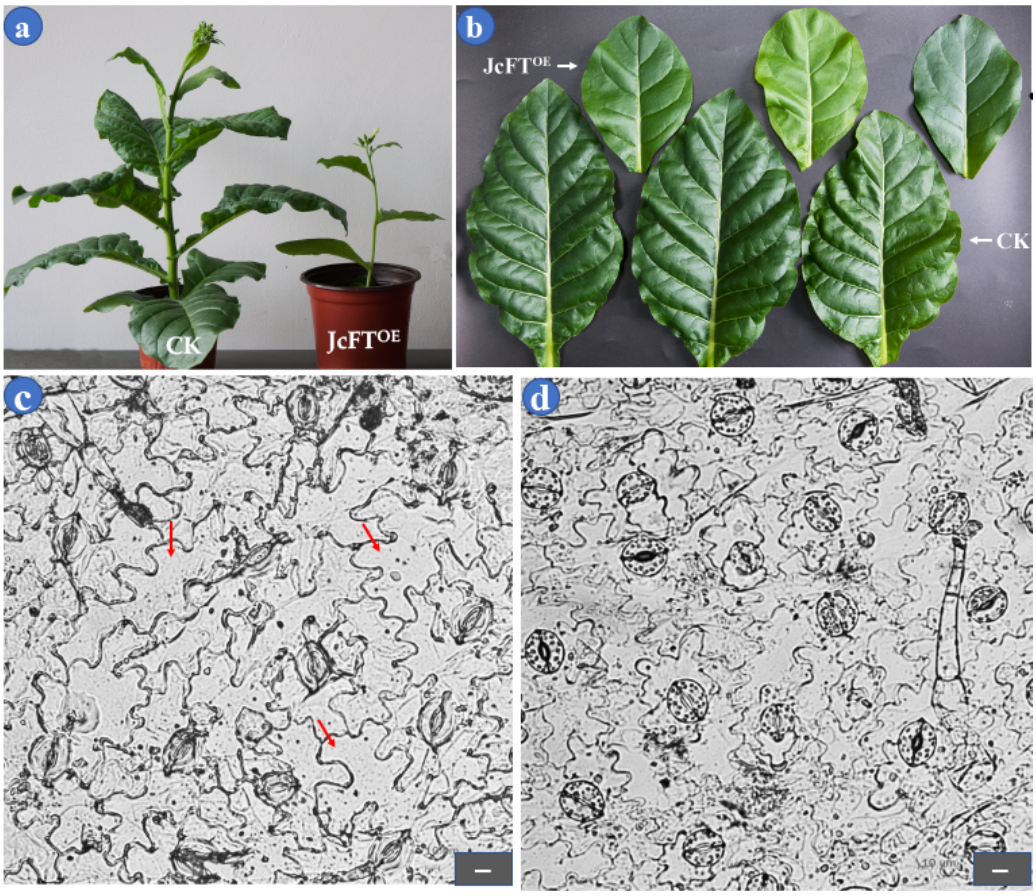

Figure S5. Comparison of leaf size and epidermal cells between JcFT^{OE} tobacco and control. (a), (b) Show the size of JcFT^{OE} tobacco and control leaves. (c) Epidermal cells of JcFT^{OE} tobacco leaves, with arrows indicating larger cell than the control. (d) Epidermal cells of control tobacco leaves. c and d showing JcFT^{OE} tobacco leaf cells were larger and fewer than the control under the same size field of view. Bar=10um)

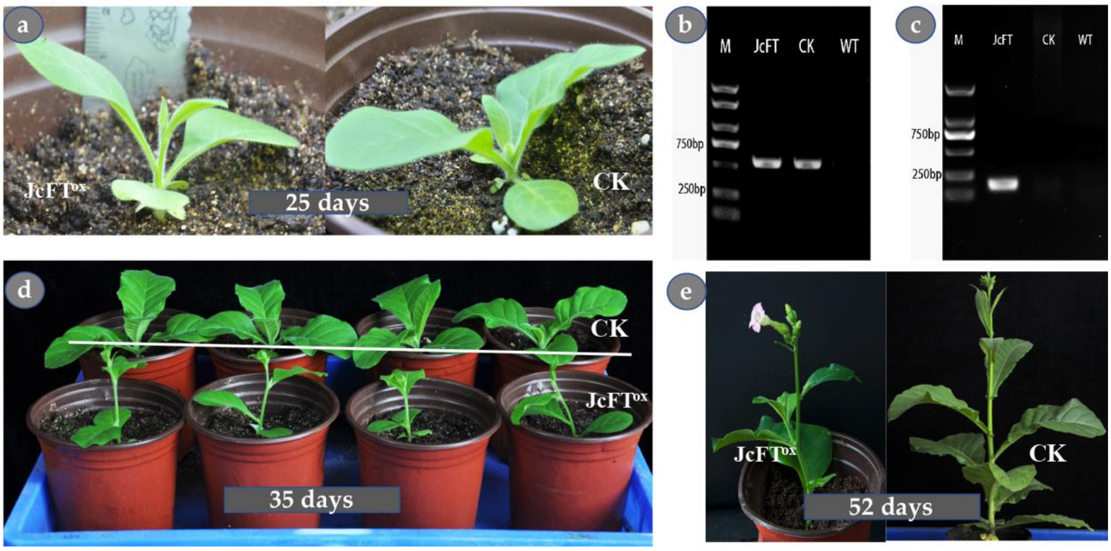

Figure S6. Characteristics and Identification of JcFT^{OE} Transgenic Plants (a, Showing the traits of JcFT^{OE} and control seedlings at 25 days after seeding; b, PCR amplification of *NPTII* using leaf DNA to identify transgenic tobacco; C, RT-PCR amplification of *JcFT* using leaf RNA to identify transgenic tobacco; d, Showing the traits of JcFT^{OE} and control seedlings at 35 days after seeding; e, Showing the traits of JcFT^{OE} and control seedlings at 52 days after seeding. CK, negative control, WT, wild-type tobacco)

2 Supplementary Tables

Table S1. The evaluation of the sequencing output data of each sample

Samples	Total Reads	Mapped Reads	Obtained Bases(bp)	Q20(%)	Q30(%)	GC(%)
CK1	42,335,870	39,937,707 (94.34%)	6,331,125,978	98.10	94.48	44.22
CK2	45,779,872	42,891,528 (93.69%)	6,835,551,846	98.13	94.53	44.27
CK3	41,733,080	39,232,507 (94.01%)	6,235,570,420	98.09	94.32	44.54
JcFT1	41,487,744	39,131,194 (94.32%)	6,200,562,974	97.99	94.21	44.29
JcFT2	49,212,278	46,432,861 (94.35%)	7,348,262,136	98.16	94.59	44.19
JcFT3	46,423,770	43,713,239 (94.16%)	6,945,825,704	97.94	94.15	44.06

Table S2. Go terms included in each group identified by ClueGO

Group name	Go terms included
regulation of jasmonic acid mediated signaling pathway	GO:0098754,GO:0006979,GO:0009611,GO:0009636,GO:0010035,GO:0016721,GO:0070887,GO:0072593,GO:1901700,GO:0080134,GO:1990748,GO:0000302,GO:0006801,GO:0033993,GO:0062197,GO:0097237,GO:1901701,GO:0000305,GO:0031347,GO:0034599,GO:0070542,GO:0098869,GO:0009753,GO:0000303,GO:0016209,GO:0034614,GO:0071398,GO:0009867,GO:0071395,GO:0071450,GO:0019430,GO:2000022,GO:0071451,GO:0004784,
response to wounding	GO:0009611,GO:0010033,GO:0010035,GO:1901700,GO:0009725,GO:0080134,GO:0000302,GO:0033993,GO:1901701,GO:0031347,GO:0070542,GO:0009753,GO:0071398,GO:0009867,GO:0071395,GO:2000022,

carbon-oxygen lyase activity, acting on phosphates	GO:0016835,GO:0008610,GO:0016838,GO:0006720,GO:0000287,GO:0008299,GO:0010333,GO:0006721,GO:0016114,GO:0016101,GO:0016102,
Detoxification	GO:0098754,GO:0009636,GO:0010033,GO:0070887,GO:0009725,GO:1990748,GO:0097237,GO:1901701,GO:0098869,GO:0016209,
oxylipin metabolic	GO:0016701,GO:0019842,GO:0051213,GO:0008610,GO:0016702,GO:0009699,GO:0034440,GO:0031407,GO:0006633,GO:0031408,
isoprenoid biosynthetic process	GO:0006629,GO:0008610,GO:0006720,GO:0008299,GO:0006721,GO:0016114,GO:0006633,
regulation of hormone levels	GO:0010817,GO:0042445,GO:0009308,GO:0009690,
oxidoreductase activity,acting on paired donors, with incorporation or reduction of molecular oxygen	GO:0004497,GO:0016705,GO:0020037,GO:0005506,
cellular response to chemical stimulus	GO:0010033,GO:0070887,GO:0009725,GO:0033993,
carboxy-lyase activity	GO:0016831
UDP-glycosyltransferase activity	GO:0008194

Table S3. Statistics of the expression level of the DEGs (differentially expressed genes) involved in auxin and cytokinin metabolic processes

Gene symbol	CK1_count	CK2_count	CK3_count	JcFT1_count	JcFT2_count	JcFT3_count	FDR[1]	log2FC (JcFT / CK)	regulated
LOC107826904	241	284	193	871	1433	928	7.51E-24	2.01636	up
LOC107764714	14	24	6	139	188	208	3.48E-17	3.45967	up
LOC107790150	253	288	256	131	173	133	8.27E-06	-1.01701	down
LOC107790677	68	102	54	880	1239	1241	7.53E-79	3.75739358	up
LOC107791003	158	172	102	738	947	913	1.04E-38	2.43982938	up
LOC107800706	26	35	27	180	167	180	1.49E-14	2.44439094	up
LOC107769258	428	541	325	826	1282	1557	2.28E-09	1.34546629	up
LOC107794372	245	253	254	702	914	1056	4.67E-24	1.67078621	up

FDR: false discovery rate; FC: fold change

Table S4. Statistics of the expression level of the DEGs (differentially expressed genes) involved in JAZs and MYC2s

Gene symbol	CK1_count	CK2_count	CK3_count	JcFT1_count	JcFT2_count	JcFT3_count	FDR[1]	log2FC (JcFT / CK)	regulated
LOC107815661	16	21	24	110	139	109	7.57E-10	2.40263701	up
LOC107808301	308	336	210	1356	1465	1480	2.73E-40	2.19082378	up
LOC107801671	0	13	9	42	64	63	0.00049604	2.79963287	up
LOC107798038	260	297	249	651	751	616	1.76E-12	1.17955451	up
LOC107782755	172	215	120	420	424	463	2.08E-07	1.22946198	up
LOC107775241	376	356	260	1139	1744	1829	1.27E-30	2.08689432	up
LOC107774268	175	231	152	751	799	899	1.69E-30	1.99250006	up
LOC107767880	992	484	867	1928	2699	2927	0.00082649	1.51092957	up
LOC107767538	584	733	512	2313	3624	3834	9.75E-44	2.25999674	up
LOC107766083	958	994	826	5079	7301	8182	1.98E-85	2.72785972	up
LOC107765893	36	94	54	235	391	324	6.90E-12	2.22183845	up
LOC107763941	275	201	194	1862	2564	3060	1.02E-72	3.3167375	up
LOC107763851	334	420	236	1948	2145	1979	3.82E-48	2.47948458	up
LOC107761668	788	633	581	1257	1567	1884	3.55E-09	1.07389206	up
LOC107761032	55	47	57	104	207	260	1.51E-05	1.6658809	up
newGene_9590	56	110	80	232	191	240	7.45E-05	1.30156695	up
NewGene_11520	59	97	51	178	204	224	7.17E-06	1.41079654	up

FDR: false discovery rate; FC: fold change

Table S5. The annotation of DEGs (differentially expressed genes) involved in the oxylipin metabolism

Gene symbol	FDR	log2FC (JcFT / CK)	Regulated	KEGG_annotation	Pfam_annotation	Swiss_Prot_annotation
LOC107813858	2.41E-05	3.64619475	up	K10529|0.0|nsy:104242207|K10529 alpha-dioxygenase [EC:1.14.99.-] | (RefSeq) alpha-dioxygenase 1	Animal haem peroxidase	Alpha-dioxygenase 1 OS=Arabidopsis thaliana OX=3702 GN=DOX1 PE=1 SV=1
LOC107763642	6.40E-07	5.36046233	up	K00454|4.13e-80|nsy:104226762|K00454 lipoxygenase [EC:1.13.11.12] | (RefSeq) linoleate 13S-lipoxygenase 2-1, chloroplastic-like	Lipoxygenase	Linoleate 13S-lipoxygenase 2-1, chloroplastic OS=Solanum tuberosum OX=4113 GN=LOX2.1 PE=1 SV=1
LOC107800317	5.85E-09	-1.3565446	down	K00454|0.0|nta:107800317|K00454 lipoxygenase [EC:1.13.11.12] | (RefSeq) linoleate 13S-lipoxygenase 2-1, chloroplastic-like	Lipoxygenase	Linoleate 13S-lipoxygenase 2-1, chloroplastic OS=Solanum tuberosum OX=4113 GN=LOX2.1 PE=1 SV=1

LOC107814671	2.43E-16	1.58194109	up	K00454|0.0|nta:107814671|K00454 lipoxygenase [EC:1.13.11.12] | (RefSeq) ACRE44; linoleate 13S-lipoxygenase 3-1, chloroplastic-like	Lipoxygenase	Linoleate 13S-lipoxygenase 3-1, chloroplastic OS=Solanum tuberosum OX=4113 GN=LOX3.1 PE=1 SV=1
LOC107771567	1.81E-65	2.37758103	up	K00454|0.0|nta:107771567|K00454 lipoxygenase [EC:1.13.11.12] | (RefSeq) linoleate 13S-lipoxygenase 3-1, chloroplastic	Lipoxygenase	Linoleate 13S-lipoxygenase 3-1, chloroplastic OS=Solanum tuberosum OX=4113 GN=LOX3.1 PE=1 SV=1
LOC107760301	0.00125839	6.1038016	up	K15718|0.0|nta:107760301|K15718 linoleate 9S-lipoxygenase [EC:1.13.11.58] | (RefSeq) linoleate 9S-lipoxygenase 6	Lipoxygenase	Linoleate 9S-lipoxygenase 6 (Fragment) OS=Solanum tuberosum OX=4113 GN=LOX1.6 PE=1 SV=1
LOC107829038	7.15E-17	2.30044224	up	K00454|0.0|nta:107829038|K00454 lipoxygenase [EC:1.13.11.12] | (RefSeq) lipoxygenase 6, chloroplastic-like	Lipoxygenase	Lipoxygenase 6, chloroplastic OS=Arabidopsis thaliana OX=3702 GN=LOX6 PE=2 SV=1
LOC107806322	0.00044675	1.22975808	up	K15718|0.0|nta:107806322|K15718 linoleate 9S-lipoxygenase [EC:1.13.11.58] | (RefSeq) Lox1; probable linoleate 9S-lipoxygenase 5	Lipoxygenase	Probable linoleate 9S-lipoxygenase 5 OS=Solanum tuberosum OX=4113 GN=LOX1.5 PE=2 SV=1
LOC107770253	1.67E-06	1.17909227	up	K15718|0.0|nta:107770253|K15718 linoleate 9S-lipoxygenase [EC:1.13.11.58] | (RefSeq) LOX; probable linoleate 9S-lipoxygenase 5	Lipoxygenase	Probable linoleate 9S-lipoxygenase 5 OS=Solanum tuberosum OX=4113 GN=LOX1.5 PE=2 SV=1

FDR: false discovery rate; FC: fold change

Table S6. The annotation of DEGs (differentially expressed genes) involved in the isoprenoid biosynthetic process

Gene symbol	FDR	log2FC (JcFT / CK)	Regulated	KEGG_annotation	Pfam_annotation	Swiss_Prot_annotation
LOC107798542	6.73E-71	5.26358189	up	K21925|7.10e-247|sot:102591915|K21925 (-)-camphene/tricyclene/(4S)-limonene/myrcene synthase [EC:4.2.3.117 4.2.3.105 4.2.3.16 4.2.3.15] | (RefSeq) (-)-camphene/tricyclene synthase, chloroplastic	Terpene synthase family, metal binding domain	(R)-linalool synthase TPS5, chloroplastic OS=Solanum lycopersicum OX=4081 GN=TPS5 PE=1 SV=1
LOC107828918	2.65E-22	1.97269472	up	K21925|6.17e-195|sly:101245212|K21925 (-)-camphene/tricyclene/(4S)-limonene/myrcene synthase [EC:4.2.3.117 4.2.3.105 4.2.3.16 4.2.3.15] | (RefSeq) TPS3; (-)-camphene/tricyclene synthase, chloroplastic	Terpene synthase family, metal binding domain	(R)-linalool synthase TPS5, chloroplastic OS=Solanum lycopersicum OX=4081 GN=TPS5 PE=1 SV=1
LOC107798543	2.00E-33	2.45172483	up	K21925|2.49e-277|sly:101245212|K21925 (-)-camphene/tricyclene/(4S)-limonene/myrcene synthase [EC:4.2.3.117 4.2.3.105 4.2.3.16 4.2.3.15] | (RefSeq) TPS3; (-)-camphene/tricyclene synthase, chloroplastic	Terpene synthase family, metal binding domain	(-)-camphene/tricyclene synthase, chloroplastic OS=Solanum lycopersicum OX=4081 GN=TPS3 PE=1 SV=1
LOC107769393	0.00092823	6.83809736	up	K18117|0.0|sot:102577550|K18117 viridiflorene synthase [EC:4.2.3.88] | (RefSeq) PVS3, VS1; putative vetispiradiene synthase 5	Terpene synthase family, metal binding domain	Viridiflorene synthase OS=Solanum lycopersicum OX=4081 GN=TPS32 PE=1 SV=1

LOC107816174	6.55E-12	3.36800565	up	K18108|5.50e-224|vvi:100232956|K18108 (-)-alpha-terpineol synthase [EC:4.2.3.111] | (RefSeq) (-)-a-terpineol synthase	Terpene synthase family, metal binding domain	(-)-alpha-terpineol synthase OS=Vitis vinifera OX=29760 PE=1 SV=1
LOC107831250	7.13E-05	7.32525089	up	K18108|3.72e-233|vvi:100232956|K18108 (-)-alpha-terpineol synthase [EC:4.2.3.111] | (RefSeq) (-)-a-terpineol synthase	Terpene synthase family, metal binding domain	(-)-alpha-terpineol synthase OS=Vitis vinifera OX=29760 PE=1 SV=1
LOC107770515	0.00118156	1.41226714	up	K15813|0.0|nta:107770515|K15813 beta-amyrin synthase [EC:5.4.99.39] | (RefSeq) beta-amyrin synthase-like	Squalene-hopene cyclase C-terminal domain	Beta-amyrin synthase OS=Solanum lycopersicum OX=4081 GN=TTS1 PE=1 SV=1
LOC107819330	2.23E-14	2.73767268	up	K15803|0.0|nta:107819330|K15803 (-)-germacrene D synthase [EC:4.2.3.75] | (RefSeq) probable terpene synthase 3	Terpene synthase family, metal binding domain	(-)-germacrene D synthase OS=Vitis vinifera OX=29760 GN=VIT_19s0014g04930 PE=1 SV=1
LOC107799408	0.00602982	-2.4618371	down	K15803|0.0|nau:109241781|K15803 (-)-germacrene D synthase [EC:4.2.3.75] | (RefSeq) 5-epi-aristolochene synthase 3	Terpene synthase family, metal binding domain	5-epi-aristolochene synthase 3 OS=Nicotiana attenuata OX=49451 PE=1 SV=1
LOC107789701	7.23E-05	4.29166814	up	K15746|1.87e-221|nta:107789701|K15746 beta-carotene 3-hydroxylase [EC:1.14.15.24] | (RefSeq) beta-carotene hydroxylase 2, chloroplastic-like	Fatty acid hydroxylase superfamily	Beta-carotene hydroxylase 2, chloroplastic OS=Capsicum annuum OX=4072 GN=CA2 PE=1 SV=2
LOC107800233	0.00211646	6.2319132	up	K14173|0.0|nta:107800233|K14173 alpha-farnesene synthase [EC:4.2.3.46] | (RefSeq) alpha-farnesene synthase-like	Terpene synthase family, metal binding domain	Alpha-farnesene synthase OS=Ricinus communis OX=3988 GN=TPS7 PE=1 SV=2
LOC107779472	1.67E-06	4.29907445	up	K14173|0.0|nta:107779472|K14173 alpha-farnesene synthase [EC:4.2.3.46] | (RefSeq) alpha-farnesene synthase-like	Terpene synthase family, metal binding domain	Alpha-farnesene synthase OS=Ricinus communis OX=3988 GN=TPS7 PE=1 SV=2

LOC107779444	0.00211583	4.4385794	up	K13789 | 1.74e-227 | nta:107779444 | K13789 geranylgeranyl diphosphate synthase, type II [EC:2.5.1.1 2.5.1.10 2.5.1.29] | (RefSeq) heterodimeric geranylgeranyl pyrophosphate synthase small subunit, chloroplastic-like	Polyprenyl synthetase	Heterodimeric geranylgeranyl pyrophosphate synthase small subunit, chloroplastic OS=Arabidopsis thaliana OX=3702 GN=GGR PE=1 SV=2
LOC107796407	0.0038812	-1.1505524	down	K01641 | 0.0 | nto:104107138 | K01641 hydroxymethylglutaryl-CoA synthase [EC:2.3.3.10] | (RefSeq) hydroxymethylglutaryl-CoA synthase-like	Hydroxymethylglutaryl-coenzyme A synthase C terminal	Hydroxymethylglutaryl-CoA synthase OS=Arabidopsis thaliana OX=3702 GN=HMGS PE=1 SV=2
LOC107766711	8.06E-05	2.29808433	up	K00869 | 2.45e-289 | nta:107766711 | K00869 mevalonate kinase [EC:2.7.1.36] | (RefSeq) mevalonate kinase-like	GHMP kinases N terminal domain	Mevalonate kinase OS=Arabidopsis thaliana OX=3702 GN=At5g27450 PE=2 SV=1
LOC107772124	0.00685795	-1.3880326	down	K00021 | 0.0 | nta:107772124 | K00021 hydroxymethylglutaryl-CoA reductase (NADPH) [EC:1.1.1.34] | (RefSeq) 3-hydroxy-3-methylglutaryl-coenzyme A reductase 1	Hydroxymethylglutaryl-coenzyme A reductase	3-hydroxy-3-methylglutaryl-coenzyme A reductase 1 OS=Solanum tuberosum OX=4113 GN=HMG1 PE=2 SV=1
LOC107770799	0.00244814	-1.1359608	down	--	--	--
LOC107772411	1.41E-06	-1.3298255	down	--	--	--

FDR: false discovery rate; FC: fold change

Table S7. DEGs (differentially expressed genes) related to the jasmonic acid reaction

Gene symbol	CK1_count	CK2_count	CK3_count	JcFT1_count	JcFT2_count	JcFT3_count	FDR	log2FC (JcFT / CK)	Regulated
LOC107798243	91	68	84	169	233	248	1.67E-05	1.2552126	up
LOC107767538	584	733	512	2313	3624	3834	9.75E-44	2.25999674	up
LOC107766083	958	994	826	5079	7301	8182	1.98E-85	2.72785972	up
LOC107761032	55	47	57	104	207	260	1.51E-05	1.6658809	up
LOC107808301	308	336	210	1356	1465	1480	2.73E-40	2.19082378	up
LOC107798038	260	297	249	651	751	616	1.76E-12	1.17955451	up
LOC107763851	334	420	236	1948	2145	1979	3.82E-48	2.47948458	up
LOC107815661	16	21	24	110	139	109	7.57E-10	2.40263701	up
LOC107801671	0	13	9	42	64	63	0.0004960	2.79963287	up
LOC107767880	992	484	867	1928	2699	2927	0.0008264	1.51092957	up
LOC107763941	275	201	194	1862	2564	3060	1.02E-72	3.3167375	up
newGene_32933	7674	8186	8798	25778	32989	17048	0.002213	1.84449	up

FDR: false discovery rate; FC: fold change

Table S8. The primers for PCR, RT-PCR and RT-qPCR

	Accession number/Gene symbol	Forward Primer (FP)	Reverse Primer (RP)	Length of PCR product (bp)	FP TM (℃)	RP TM (℃)	FP GC%	RP GC%
NPTII	X57709	GGAGAGGCTATTCGGCTATGAC	CACCATGATATCGGCAAGCAG	547	59	58	55	50
JcFT	NM_001308752	CTAGCCCAAGTGACCCTAATCTC	GGGCTCTCATAGCACACTATCTC	109	58	58	52	52
EF-1a	AF120093	TGAGATGCACCACGAAGCTC	CCAACATTGTCACCAGGAAGTG	51	59	59	55	50
L25	L18908	CCCCTCACCACAGAGTCTGC	AAGGGTGTTGTTGTCCTCAATCTT	51	62	59	65	42
DEGs of cytokinin metabolic process	LOC107790677	CGCATGTCACAGTCTATTTTGAG	TAACCCAAGCGGAACCATGC	179	58.11	60.96	43.48	55
	LOC107791003	AGTGACACCAGAGGAGGAGG	ATGCCGACCTCAGAAATCCC	53	60.25	59.82	60	55
	LOC107800706	AGAGCATCAGGCGTTCAACA	TGTGTGCACTGTGGAGCATT	104	59.96	60.47	50	50
	LOC107769258	GGGCCCCTTTGTCAATTGTT	GAAGCCAACTAAAGACTGGGGA	137	58.94	59.96	50	50
	LOC107794372	CCACGAACGAGTTGCTTCCA	TTTCACCGCGCCATTTCTTG	81	60.88	60.04	55	50
DEGs of auxin metabolic process	LOC107826904	GTGCTATGTGGATGGCTAACTC	AAGCACTCATCTTCGCCGTG	199	58.87	61.02	50	55
	LOC107764714	AACAATACGCAGAAGTTTTTCCCT	CAAGCACTCATCATCGCCG	135	59.42	59.36	37.5	57.89
	LOC107790150	TGGGAGCGACAAATCCAAGG	GCACCCGAGTTAGGGATTCA	143	60.32	59.46	55	55
DEGs of JAZ	LOC107815661	TACTACTGGTAGACGGGGAGC	CCACCCGTGACTTCGTGAT	72	60.13	59.71	57.14	57.89
	LOC107808301	GGAAAGATAGGGCAACGGCT	TCGCCAACCAAAAAGGTTCAT	180	60.11	58.96	55	42.86
	LOC107801671	TTTGGTTTTATTAGTTGCTACCGGA	GTGACCTTGCCGGAATCTACA	70	59	60.07	36.00	52.38
	LOC107798038	TGGTTGGAATTGGGTGCTCA	TGAATAACCATCTCTCTGGTTTCA	223	59.81	57.63	50.00	37.50

	LOC107775241	GACCTGTTGGGGCGACTAC	TGCAGCGTTCATCACCCTT	107	60.08	59.93	63.16	52.63
	LOC107767880	ATGGACCTCAACACCAGACC	AATGGCCACTTCATCCCAGA	74	59.31	59	55.00	50.00
	LOC107767538	TCTTCTACGGTGGGACGGT	TGGTTCACACAAGTGCCGT	172	59.92	60.08	57.89	52.63
	LOC107766083	AGAAATCGGGTGAGTGCTCC	TGGTTTATGGCAATAGGCTGC	137	59.75	58.97	55.00	47.62
	LOC107763941	ATGCTAAAGGAGCACTGGCT	TCCAAAAATCGGGCAAGGGT	55	59.38	60.18	50.00	50.00
	LOC107763851	CACATCTGCTGCTGCCACT	CTGGTGCTGTACTTGCGGT	175	60.67	60.3	57.89	57.89
	LOC107761032	TTGCTTTCCGCCCCTCATT	GGGATAAACAACATCACCTCTGTC	124	59.92	59.36	52.63	45.83
DEGs of AUX/IAA, SAUR, GH3, ARG	LOC107806659	AACAAGACTCAGCCCCAGC	AGGTGCTCCATCCATGCTAAC	146	59.93	60.13	57.89	52.38
	LOC107764405	GGTGGCGGTGGAGAATTAAGA	GGCTTGGCTGAGTCCTTGTT	185	60.07	60.54	52.38	55
	LOC107830653	CGTGGGAGATGTACCTTGGC	CAACATTTTCAGCTCCTGCTCT	129	60.46	59.18	60.00	45.45
	LOC107813771	TCCCTGTAGCGTGGATGTCT	GTCTACCTAGTCAGAGGGTGGT	199	60.32	60.03	55.00	54.55
	LOC107783912	ATGGGTGGTCTCACAATTCCA	TTTCTTCCTCGCCTGAACTGTA	159	59.29	59.37	47.62	45.45
	LOC107782902	CTGAGTTGCCATTTGGCCTAT	GAGGCATACGAATAGCCATGA	172	58.62	57.68	47.62	47.62
	LOC107763329	CCCCACAGCACTCAGCAAAA	GGCATACGGATAGCCATAGTCG	137	61.11	60.48	55.00	54.55
	LOC107832378	CCTACACACTCCACATGCCTT	GAGGGAGTATTTATGGAGGGAGAC	55	60	59.47	52.38	50
	LOC107794774	GCCAGTTCCCTTATCTCAAGTCA	GAGTCTAGGGGTCTTCCCGT	133	60.06	60.03	47.83	60.00
	LOC107795884	GCGCACCTACTGTGCATGA	GTGACGCCCTGCTTATGTGT	130	60.74	60.67	57.89	55.00
	LOC107771982	TTGGGGTATGCTAAAGTGCGT	AAGGGCTGCTAACAACAAAGC	70	60	59.66	47.62	47.62
DEGs involved in oxylipin	LOC107813858	ACTTTCGCGTTCCTCCACAG	GGCTGACCACCCAATCGTT	299	60.6	60.3	55	57.89
	LOC107763642	AAGCGAGAAAGGTACTCCCG	TAGCATCTGGACAACACGAC	181	59.47	57.64	55	50
	LOC107814671	GCACCAAAATGAAAACCCCAC	AGTACGGTTGCTCTCACCTT	73	58.78	58.66	47.62	50
	LOC107771567	CCTCCGAAATGCAGAGTGGT	ATCTACGCATGAGAGGTGGC	164	60.04	59.61	55	55

metabolic process	LOC107760301	AACGTGGAACATAGATTTGGCT	CGGCGCTAAATAAGGGAGAGT	163	58.31	59.93	40.91	52.38
	LOC107829038	CGAGGACATTGCCAGCACTA	AAACCGGGCTACCGATGATG	58	60.11	60.18	55	55
	LOC107806322	TTTCCTAGTTAGAAACTTCACAACACA	CCCTCCGACCAAGGAACTCA	264	59.17	61.19	33.33	60
	LOC107770253	TGTGTGAAACAAGGAGAAGACCAAG	TCCAGCATCTCTTACCTCCAAT	218	61.38	58.61	44	45.45
DEGs involved in isoprenoid biosynthetic process	LOC107831250	GACAGATTGGTGGAGGGCTT	TGCTGTGAGGAGTACAGACATC	105	59.67	59.51	55	50
	LOC107769393	TGTTCAGTTCTCTGCACCAAGA	AAGCAAGATAAAGAGCTAGGGTT	75	59.83	57.56	45.45	39.13
	LOC107800233	CGTGAAAACTAAGTCCAGCG	CCTGAGAAGCCTGAAGCACA	70	57.14	59.96	50	55
	LOC107798542	GAGGTGGTGGAAGAGCACAT	CCATCTCGTCAAGACTGCCA	194	59.67	59.75	55	55
	LOC107779444	AGGTGAGGCCAATTGCTAGTC	CGAGCTAGGCACGAAAAGGTA	125	60.07	60.14	52.38	52.38
	LOC107779472	CGGCCTATTCACTTTCGTCC	TTACAAGTATGCCAGACCACCA	54	58.71	59.36	55	45.45
